# Supplementary material for: P-TRAP: a Panicle Trait Phenotyping tool
Source: BMC Plant Biol. 2013 Aug 29;13:122. doi: 10.1186/1471-2229-13-122 (PMC3848748; doi:10.1186/1471-2229-13-122)
Supplement: Additional file 7 — P-TRAP source code. The Netbeans project that contains the source code of the application. [file 1471-2229-13-122-S7.zip › javadoc.zip!/javadoc/org-bioalg-widgetfactory/org/bioalg/widgetfactory/WidgetPopUpMenuHandler.html]

WidgetPopUpMenuHandler (WidgetFacory)


JavaScript is disabled on your browser.


- Package
- Class
- Tree
- Deprecated
- Index
- Help

*org.bioalg.widgetfactory 1.0*

- Prev Class
- Next Class

- Frames
- No Frames

- All Classes

- Summary:
- Nested |
- Field |
- Constr |
- Method

- Detail:
- Field |
- Constr |
- Method


org.bioalg.widgetfactory

## Class WidgetPopUpMenuHandler

- java.lang.Object
- - org.bioalg.widgetfactory.WidgetPopUpMenuHandler

- All Implemented Interfaces:
  :   java.awt.event.ActionListener, java.util.EventListener, org.netbeans.api.visual.action.PopupMenuProvider

  ---

    

  ```
  public final class WidgetPopUpMenuHandler
  extends java.lang.Object
  implements org.netbeans.api.visual.action.PopupMenuProvider, java.awt.event.ActionListener
  ```

  This is a general widget's pop up menu handler. The commands of the menu are
  dealt with according to their names.

  Author:
  :   faroq

- - ### Nested Class Summary

    Nested Classes

    | Modifier and Type | Class and Description |
    | --- | --- |
    | `static class` | `WidgetPopUpMenuHandler.WIDGET_COMMANDS` Command types. |
  - ### Constructor Summary

    Constructors

    Constructor and Description

    `WidgetPopUpMenuHandler(org.netbeans.api.visual.graph.GraphScene masterScene)`

    `WidgetPopUpMenuHandler(org.netbeans.api.visual.graph.GraphScene masterScene,
    java.util.ArrayList<WidgetPopUpMenuHandler.WIDGET_COMMANDS> commands)`
  - ### Method Summary

    Methods

    | Modifier and Type | Method and Description |
    | --- | --- |
    | `void` | `actionPerformed(java.awt.event.ActionEvent ae)` |
    | `java.util.ArrayList<WidgetPopUpMenuHandler.WIDGET_COMMANDS>` | `buidlallcommands()` |
    | `java.awt.Color` | `getColorBycommand(WidgetPopUpMenuHandler.WIDGET_COMMANDS c)` |
    | `WidgetPopUpMenuHandler.WIDGET_COMMANDS` | `getcommandByColor(java.awt.Color c)` |
    | `javax.swing.JPopupMenu` | `getPopupMenu(org.netbeans.api.visual.widget.Widget widget, java.awt.Point point)` |
    | `void` | `setupCommands(java.util.ArrayList<WidgetPopUpMenuHandler.WIDGET_COMMANDS> commands)` Set up the commands of the menu. |

    - ### Methods inherited from class java.lang.Object

      `clone, equals, finalize, getClass, hashCode, notify, notifyAll, toString, wait, wait, wait`

- - ### Constructor Detail


    - #### WidgetPopUpMenuHandler

      ```
      public WidgetPopUpMenuHandler(org.netbeans.api.visual.graph.GraphScene masterScene,
                            java.util.ArrayList<WidgetPopUpMenuHandler.WIDGET_COMMANDS> commands)
      ```


    - #### WidgetPopUpMenuHandler

      ```
      public WidgetPopUpMenuHandler(org.netbeans.api.visual.graph.GraphScene masterScene)
      ```
  - ### Method Detail


    - #### getPopupMenu

      ```
      public javax.swing.JPopupMenu getPopupMenu(org.netbeans.api.visual.widget.Widget widget,
                                        java.awt.Point point)
      ```

      **Specified by:**
      :   `getPopupMenu` in interface `org.netbeans.api.visual.action.PopupMenuProvider`


    - #### getcommandByColor

      ```
      public WidgetPopUpMenuHandler.WIDGET_COMMANDS getcommandByColor(java.awt.Color c)
      ```


    - #### getColorBycommand

      ```
      public java.awt.Color getColorBycommand(WidgetPopUpMenuHandler.WIDGET_COMMANDS c)
      ```


    - #### actionPerformed

      ```
      public void actionPerformed(java.awt.event.ActionEvent ae)
      ```

      **Specified by:**
      :   `actionPerformed` in interface `java.awt.event.ActionListener`


    - #### buidlallcommands

      ```
      public java.util.ArrayList<WidgetPopUpMenuHandler.WIDGET_COMMANDS> buidlallcommands()
      ```


    - #### setupCommands

      ```
      public void setupCommands(java.util.ArrayList<WidgetPopUpMenuHandler.WIDGET_COMMANDS> commands)
      ```

      Set up the commands of the menu.

      Parameters:
      :   `commands` - An `ArrayList` of `WidgetPopUpMenuHandler.WIDGET_COMMANDS`.


- Package
- Class
- Tree
- Deprecated
- Index
- Help

*org.bioalg.widgetfactory 1.0*

- Prev Class
- Next Class

- Frames
- No Frames

- All Classes

- Summary:
- Nested |
- Field |
- Constr |
- Method

- Detail:
- Field |
- Constr |
- Method
